# Qualitative evaluation of trace elements in commercially packaged forms of tobacco using laser-induced breakdown spectroscopy

**DOI:** 10.1038/s41598-024-72619-1

**Published:** 2024-09-14

**Authors:** A. Kripa Adlene Edith, Ravikiran Ongole, V. K. Unnikrishnan, U. K. Adarsh

**Affiliations:** 1 Department of Oral Medicine and Radiology, Manipal College of Dental Sciences Mangalore, Manipal Academy of Higher Education, Manipal, 576104 Karnataka India; 2 Department of Atomic Molecular Physics, Manipal Academy of Higher Education, Manipal, 576104 Karnataka India

**Keywords:** Oral cancer, Smokeless tobacco, Trace elements, Laser-induced breakdown spectroscopy, Oncology, Risk factors, Engineering, Optics and photonics, Physics

## Abstract

Oral cancer is the most common malignancy in many developing countries, such as India, due to increased consumption of smokeless tobacco. The trace elemental components in commercially packaged forms of tobacco can play a significant role in the pathogenesis of oral cancer. To qualitatively assess the trace elements in various types of commercially packaged forms of tobacco using laser-induced breakdown spectroscopy (LIBS). Two popular varieties of ‘Paan masala’ that contained a mixture of slaked lime with areca nut, catechu, and other flavouring agents (tobacco was absent) and four types of packaged tobacco were obtained from ‘Paan’ shops. The contents in the packets were made into pellets using a hydraulic press and subjected to elemental analysis using LIBS. A ten-trial experiment was carried out on all six pellets. The National Institute of Standards and Technology (NIST) database was used to assess the emission lines. The elements obtained from commercially packaged tobacco and Paan masala were similar: calcium (Ca), iron (Fe), aluminium (Al), nickel (Ni), and chromium (Cr). Substances that cause DNA damage and carcinogenesis are inorganic elements such as nickel. Our study revealed that carcinogens such as nickel are present in the commercially packaged forms of tobacco and ‘Paan masala’ samples.

## Introduction

Oral potentially malignant disorders (OPMDs) are defined as “any oral mucosal abnormality that is associated with a statistically increased risk of developing oral cancer”. These are diseases with various clinical features, histological classifications such as hyperkeratosis and different grades of dysplasia, and a mixture of risk factors or etiology. OPMDs are classified as oral leukoplakia, oral erythroplakia, proliferative verrucous leukoplakia, oral lichen planus, and oral submucous fibrosis (OSMF)^1,2^. Leukoplakia and OSMF have a prevalence of 4.11% and 4.96% respectively globally with the highest incidence in the Asian population and its common risk factors are tobacco and betel quid chewing along with alcohol consumption^3^. It is well established that tobacco in both smokeless and smoked forms plays a major role in causing oral potentially malignant disorders and oral cancer^4^. The end products of unburned tobacco are sucked, chewed (dipped), gargled, applied to the gums or teeth, and sometimes fine tobacco mixtures are inhaled into the nostril refers to as smokeless tobacco^5^. It is also often referred to as “spit” or “spitting” tobacco, as chewers spit saliva and extracts of tobacco that accumulate in the oral cavity. This smokeless tobacco usually contains nicotine, tobacco, abrasives, chemicals, sweeteners, and salts. These habits influence oral health and increase the risk of oropharyngeal carcinoma^6^. The use of smokeless tobacco with or without betel quid is common in many South Asian countries. Smokeless tobacco is used by 26% of the total Indian adult population and is out of proportionally consumed by the middle- and lower-income population^7^. The reason to consume smokeless tobacco is the belief of less injurious compared to other forms of tobacco, relief from anxiety and toothache, family traditions, lack of awareness, and addiction^8^. Addiction and dependence on smokeless tobacco is the impact of nicotine in the dopaminergic system which assists in reinforcing and rewarding conduct^9^. Betel quid is a mixture of substances comprising at least one of the two essential constituents such as tobacco or areca nut in raw, manufactured, or processed form wrapped together in a betel leaf and placed in the mouth or chewed which remains in contact with the mucosa for prolonged period^10,11^. On the other hand, ‘paan masala’ is a dehydrated, nonperishable preparation of areca nut, catechu (acacia catechu), slaked lime (calcium oxide and calcium hydroxide), cardamom, and a variety of artificial fragrances and flavour^12^. ‘Paan masala’ is often substituted for smokeless tobacco because it claims to contain no tobacco-related products. Areca nut is the major raw ingredient of paan masala and betel quid, which constitutes of tannins (stimulant and alkaloid). It provides relaxation, and concentration, diminishes hunger and hence these are widely consumed in lower economic populations^13^. Prolonged exposure to these products causes micromolecular alterations, which are often followed by morphological alterations in the oral mucosa that are clinically evident as OPMDs, which later may have the potential to turn into oral cancer^14^. The predisposing changes in the oral mucosa of oral cancer patients are referred to as OPMDs which have a statistically increased risk of progressing into malignancies and trace elements play a major role in causing OPMDs^15^. Trace elements are broadly classified as macro elements (Calcium (Ca), phosphorus (P), magnesium (Mg), sodium (Na), potassium (K), chlorine (Cl) and sulphur (S)) and microelements (Iron (Fe), copper (Cu), iodine (I), manganese (Mn), zinc (Zn), molybdenum (Mo), cobalt (Co), fluorine (F), selenium (Se) and chromium (Cr)). While Nickel (Ni), cadmium (Cd), arsenic (As), and lead (Pb) are considered carcinogens^16^. The process of carcinogenesis begins with damage to the DNA followed by gene mutation due to replication of the damaged DNA that causes alteration in proteins and cancer development. In India, 7% of lesions with severe epithelial dysplasia undergo malignant transformation and the mechanism involved in this malignant progression is still unknown^17^. There is a need to assess the presence of trace elements in the commercially packaged form of tobacco and paan masala to understand the role of trace elements in the causation of OPMDs and oral cancer. Our objective was to assess the presence of trace elements in commercially packaged tobacco and paan masala using laser-induced breakdown spectroscopy (LIBS).

It is well established via various experimental-analytical procedures that the metal contents in the plant species share similar qualitative composition and differ only in terms of concentrations at which they are present^18^, which is influenced by multiple physical and environmental factors^19^. As already mentioned, certain trace elements/heavy metals, like nickel, which are known to induce biological changes upon excess ingestion/consumption are also present in these samples. Thus, it is important to monitor the presence/content (qualitative/quantitative) of these elements in food and other means of human consumption. It is true that the ‘carcinogen-marked’ heavy metals are found in many of the plant materials that are often part of the human diet. However, the instance of tobacco and paan masala is different compared to other products. Since the contact and absorption in such cases with the said carcinogenic elements are direct either through inhalation or extended chewing and in both cases, the frequency of consumption is much higher compared to other food products due to addiction. The objective of the article is to report the presence of these heavy metals in the packaged tobacco/non-tobacco claimed products that should be considered by the policymakers regarding the regulations on the sale and consumption of these products.

## Materials and methods

### Sample collection

**Fig. 1:**
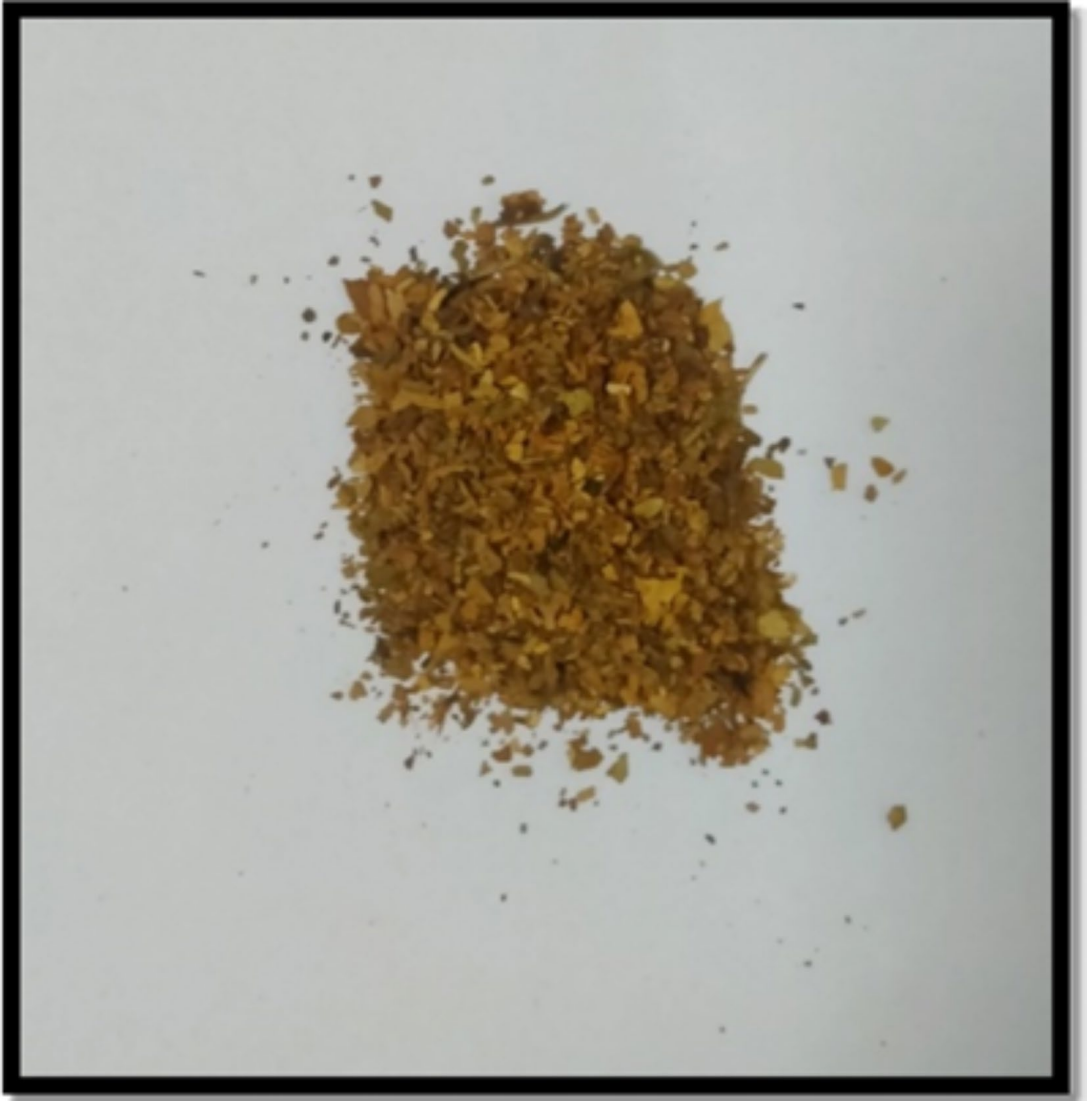
Commercially packaged form of tobacco (sample T4).

A total of four commercially packaged forms of tobacco and two paan masala packets were obtained from paan shops in Mangalore, India. The list of the ingredients as described by the manufacturers was noted. The ingredients mentioned on the ‘Paan masala’ packets were areca nuts, catechu, lime, permitted spices, cardamom, lime, menthol, and added flavours. The packets clearly mentioned that they did not contain tobacco. All commercially packaged forms of tobacco mentioned the presence of 100% tobacco. The sample names of each ‘paan masala’ and the commercially packaged form of tobacco (for the purpose of the study) were denoted as PM1, PM2, T1, T2, T3, and T4, respectively (Fig. 1).

### Sample preparation

**Fig. 2:**
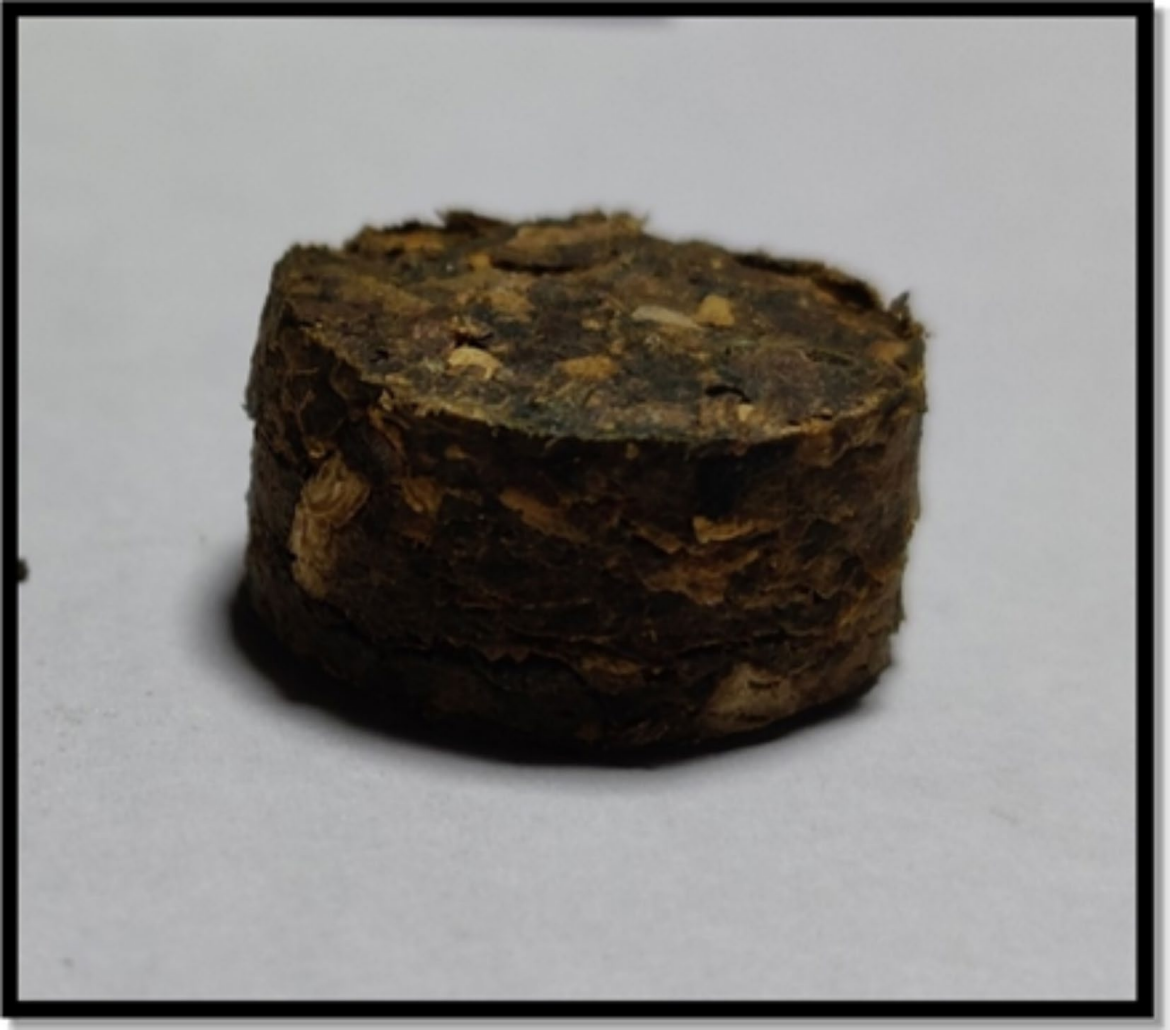
Formed pellet of sample T4.

The contents in the packet were in a coarse semi powdered form. The contents of the packet were ground, and 2 g of the powder was weighed and made into a pellet using a hydraulic press. The formed pellet (Fig. 2) was further dried using a desiccator. The obtained sample was subjected to laser-induced breakdown spectroscopy (LIBS).

LIBS is a qualitative/semiquantitative elemental characterization technique based on atomic emissions from samples. The LIBS system employed in the current study uses a high-energy pulsed laser (Nd: YAG) operating at 532 nm with a 6 ns pulse width and a 10 Hz repetition rate to ablate the sample. The laser beam was reflected in the orthogonal direction using a glass prism, followed by a biconvex lens (lens 1) with a 6 cm focal length, which focused the beam to a micrometer-size spot on the sample surface. A high-energy laser beam focused on the sample surface creates sufficient energy density to induce ablation of the sample in a localized spot, and a plasma is created. Plasma emits the characteristic emissions of its constituents, which are collected and collimated by the same lens (lens 1) used for focusing the laser. The collimated signal is then focused on to the spectrograph input by another biconvex lens (lens 2) with a focal length of 20 cm. The spectrograph grating disperses the signal in the wavelength domain, which is then recorded using a charged couple device (CCD) detector connected to a personal computer (PC). An exposure time of 10 ms is required to record each spectrum in single-shot mode, where a single pulse from the laser is used to induce and excite the plasma and then collect the corresponding plasma emissions. The spectra recorded from the samples were subjected to data preprocessing, including baseline correction and normalization. It is followed by analysis based on the known-element database (local and NIST database) to qualitatively characterize the samples. From each pellet sample, 10 spectra were recorded from different spots on the pellet to ensure the reproducibility of the identified emissions.

## Results

Two different samples of paan masala and four different samples of commercially packaged tobacco were labelled PM1, PM2, T1, T2, T3, and T4, respectively. The LIBS data were collected in the spectral range of 260–520 nm. Ten prominent emission lines from calcium were identified in the analysed spectral range at 316.17 nm, 370.71 nm, 393.75 nm, 396.26 nm, 422.68 nm, 430.53 nm, 431.03 nm, 432.15 nm, 442.76 nm, and 445.75 nm. Nine emission lines of iron were identified from the spectrum, at 285.56 nm, 318.19 nm, 344.05 nm, 358.39 nm, 360.16 nm, 373.72 nm, 400.04 nm, 404.47 nm, and 438.64 nm. Aluminium emissions were detected at 279.86 nm and 309.59 nm. The analysed region contained the prominent emission line from nickel at 280.6 nm. Like nickel, chromium emission was also prominent at a single position at 357.52 nm.

**Fig. 3:**
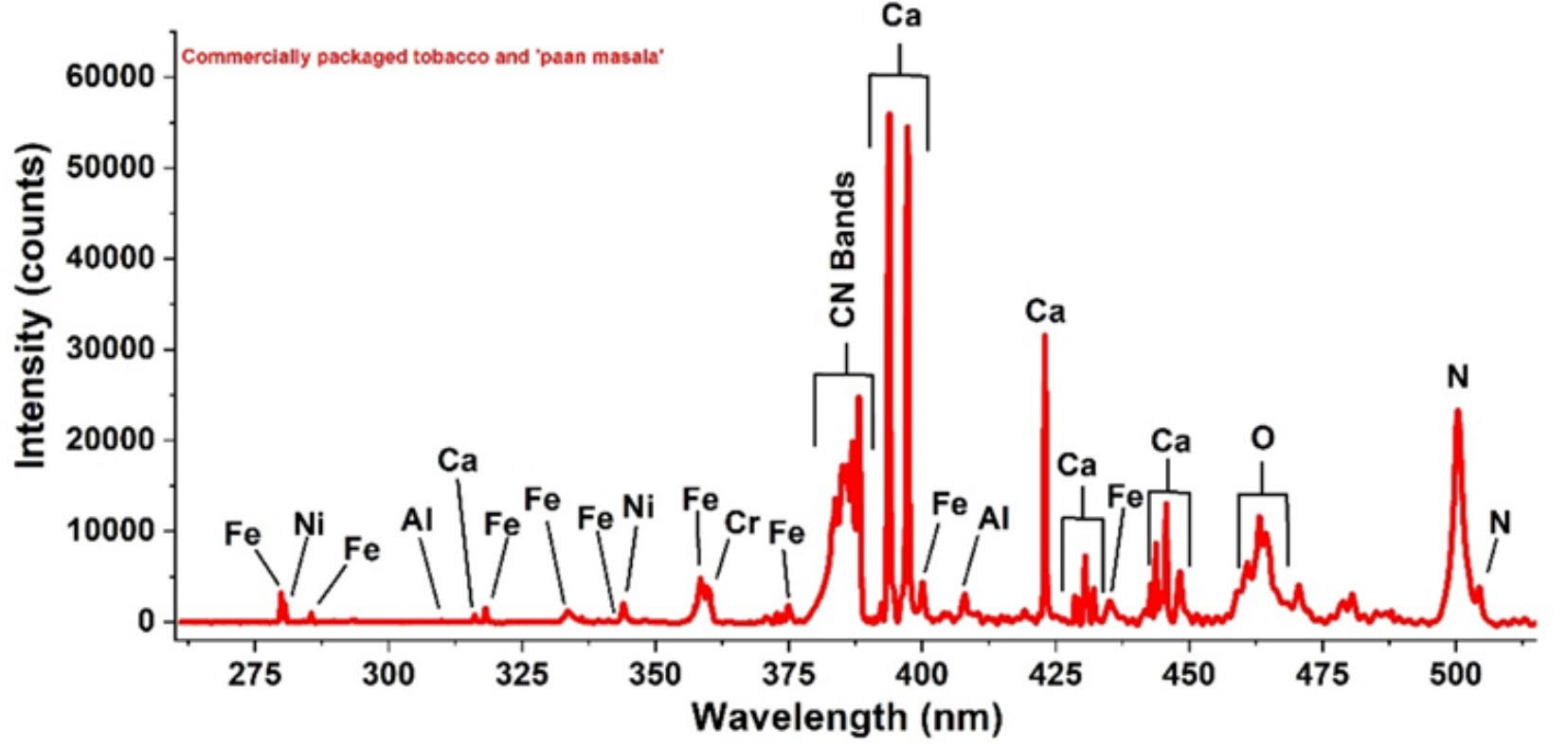
Trace elements recognized using the NIST database and characteristics lines are marked.

There was no difference in the elemental composition between the groups of ‘Paan masala’, which claimed no tobacco, and those of commercially packaged tobacco (100% tobacco). Hence, the analysed spectral range of both groups was plotted in a single graph, as shown in Fig. 3.

**Fig. 4:**
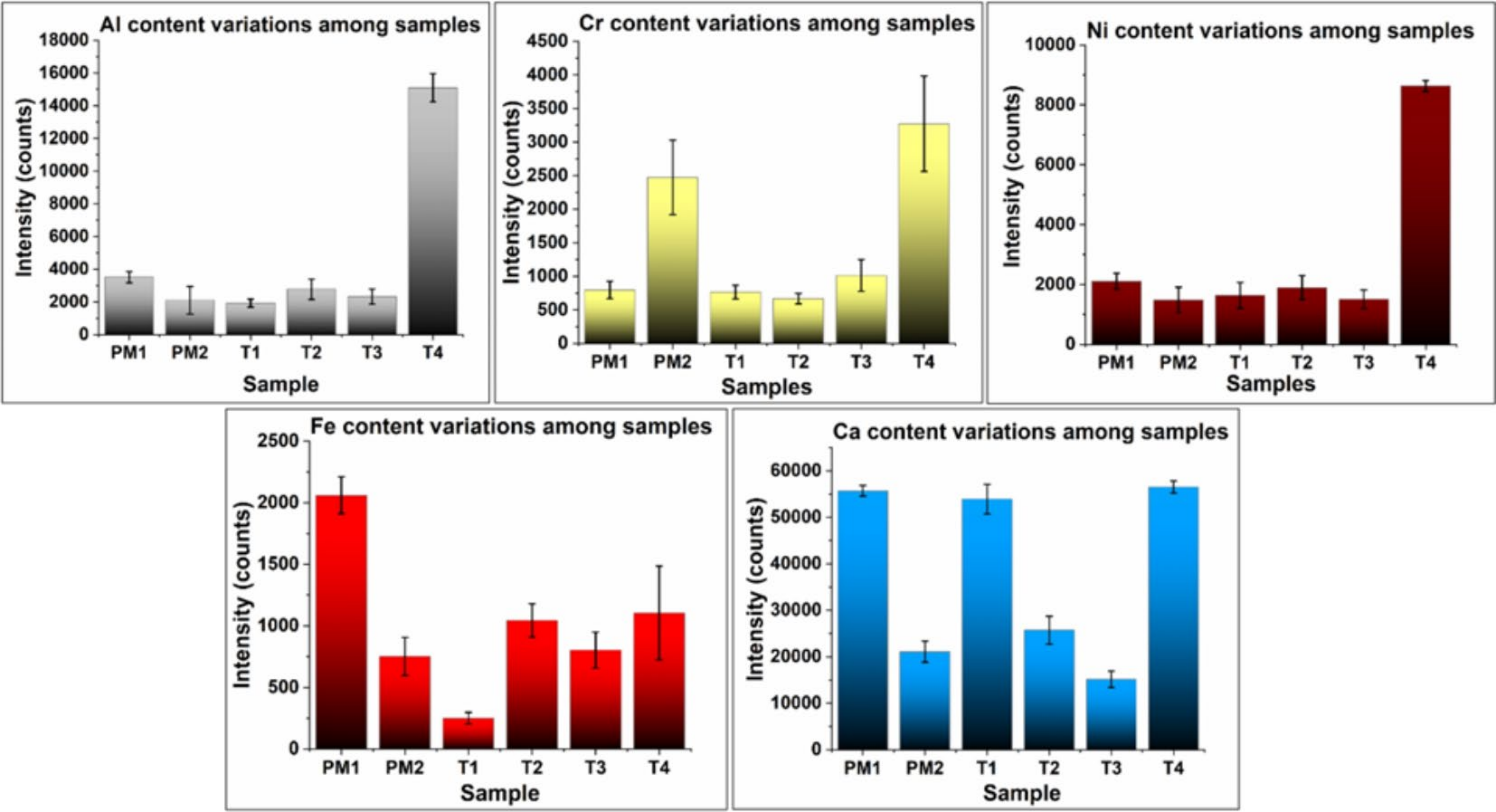
Comparison of variation of trace elements among the analysed samples.

The absolute intensity values in the LIBS spectrum do not correlate with the concentrations of the respective elements identified unless a calibration plot is constructed with known concentrations of the elements in the samples. Thus, the results of the current study are limited to only a qualitative assessment. The absolute intensity among the analysed samples is tabulated in Tables 1, 2, 3, 4, 5 and 6. However, the relative intensity variations of elements between different samples of comparable composition can indicate the relative variations in concentrations of the respective elements as shown in Fig. 4.

**Table 1:** Average intensity of trace elements present in PM1.

Elements	Most intense line (nm)	Average intensity (counts)
Al	279.86	3517.75 ± 342.8
Ni	280.62	2107.22 ± 267
Cr	357.51	797.66 ± 128.2
Fe	374.97	2061.4 ± 149.8
Ca	393.88	55702.7 ± 1162.1

**Table 2:** Average intensity of trace elements present in PM2.

Elements	Most intense line (nm)	Average intensity (counts)
Al	279.86	$\:2105.83\pm\:845.7$
Ni	280.62	$\:1481.6\pm\:427.1$
Cr	357.51	$\:2473\pm\:555.1$
Fe	374.97	$\:751.8\pm\:153.9$
Ca	393.88	$\:21092.4\pm\:2288.5$

**Table 3:** Average intensity of trace elements present in T1.

Elements	Most intense line (nm)	Average intensity (counts)
Al	279.86	$\:1931.74\pm\:247.6$
Ni	280.62	$\:1639\pm\:430$
Cr	357.51	$\:765.6\pm\:102$
Fe	374.97	$\:250.83\pm\:46.4$
Ca	393.88	$\:53918.4\pm\:3175.6$

**Table 4:** Average intensity of trace elements present in T2.

Elements	Most intense line (nm)	Average intensity (counts)
Al	279.86	$\:2776.75\pm\:623.5$
Ni	280.62	$\:1897.75\pm\:401.1$
Cr	357.51	$\:667.5\pm\:77.2$
Fe	374.97	$\:1043.67\pm\:134.7$
Ca	393.88	$\:25718.5\pm\:2989.3$

**Table 5:** Average intensity of trace elements present in T3.

Elements	Most intense line (nm)	Average intensity (counts)
Al	279.86	$\:2328.5\pm\:454$
Ni	280.62	$\:1507.4\pm\:312$
Cr	357.51	1014$\:\pm\:\:235.3$
Fe	374.97	802.85$\:\pm\:145.2$
Ca	393.88	$\:15131.3\pm\:1771.3$

**Table 6:** Average intensity of trace elements present in T4.

Elements	Most intense line (nm)	Average intensity (counts)
Al	279.86	$\:15100.7\pm\:8631.4$
Ni	280.62	$\:8631.4\pm\:181.4$
Cr	357.51	$\:3272.67\pm\:\:709.3$
Fe	374.97	$\:1105\pm\:380.3$
Ca	393.88	$\:56523.4\pm\:1303.7$

LIBS analysis of packaged tobacco products indicated the presence of 5 trace elements, namely, Al, Cr, Ni, Fe, and Ca.

### Interpretation of the statistical analysis

**Fig. 5:**
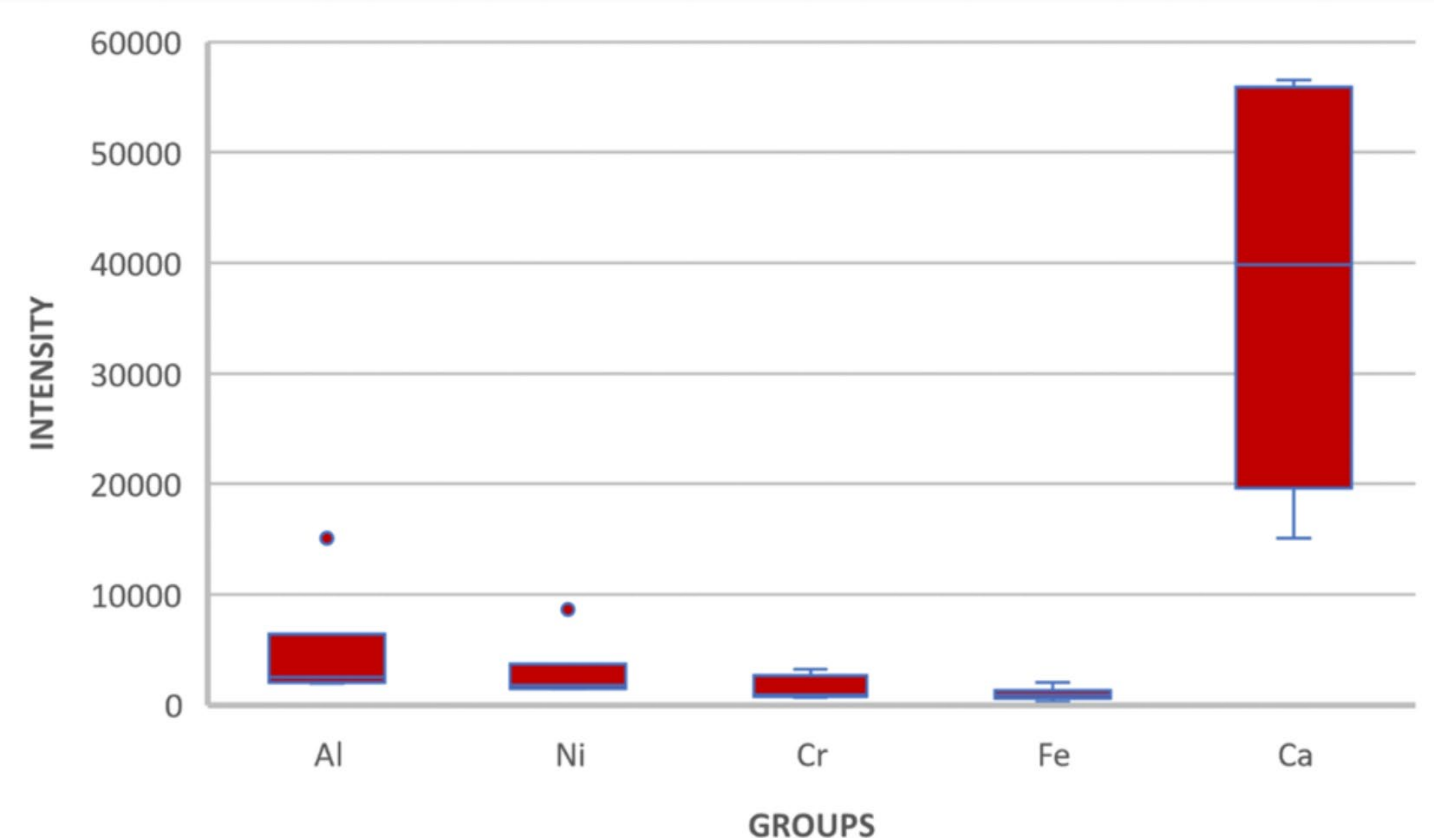
Kruskal Wallis test to compare the relative spectral intensity of trace elements between commercially packaged forms of tobacco.

The average relative intensities of the trace elements (Al, Fe, Cr, Ni, and Ca, ) in all six groups of ‘paan masala’ and commercially packaged forms of tobacco were compared using the ANOVA Kruskal Wallis test (Fig. 5). There was a statistically significant difference in the relative intensities of each trace element between different commercially packaged forms of tobacco (p-value < 0.001) that is tabulated in Table 7.

**Table 7:** Kruskal Wallis test to compare the relative spectral intensity of trace elements between commercially packaged forms of tobacco.

Elements	Intensity	Median	Interquartile range	*P* value
Al	$\:4626.88\pm\:\:2107.57$	2552.62	4351.17	< 0.001
Ni	2877.39$\:\pm\:$ 1154.99	1768.37	2237.31	< 0.001
Cr	$\:1498.40\pm\:\:449.10$	905.83	1931.84	< 0.001
Fe	$\:1002.59\pm\:\:245.02$	923.26	717.54	< 0.001
Ca	$\:38014.38\pm\:\:7894.27$	39818.45	36306.05	< 0.001

## Discussion

Our study aimed to assess elements in commercially packaged forms of tobacco and ‘paan masala’ that claimed to contain no tobacco. To the best of our knowledge, laser-induced breakdown spectroscopy (LIBS) has not been used to assess the elemental composition of commercially packaged tobacco and paan masala. Since LIBs are a “put and play” method, they have been used for various applications, such as elemental mapping of trace elements in different hard and soft tissues, leading to early diagnosis of malignancies, calculi/stones, biological or dental material such as pellets or powders, and detection of bacteria/viruses in bioaerosols^20,21^. LIBS aids in the multifaceted detection of elements and does not require sample preparation. Furthermore, it is a noncontact experimental procedure, and all states of matter, such as solid, liquid, and gas, can be assessed^22^.

It is well established that smokeless tobacco products play a major role in causing OPMDs and oral cancer^12^. Chiba et al.^23^ reviewed various toxic elements in tobacco and smokeless tobacco. Various elements, such as Cr, Al, Ni, As, Mn, Cu, Pb, mercury (Hg), and Zn, were detected in the tobacco products. Some trace elements are chemical carcinogens that may be genotoxic or nongenotoxic. Genotoxic carcinogens interact directly with DNA, resulting in chromosomal aberrations and DNA damage. The latter are chemicals that act directly as tumour promoters, induce an inflammatory response, and cause immunosuppression and tissue toxicity. The oxidation states of Cr, Be (beryllium), Ni, Cd, and As are considered carcinogens by the International Agency for Research on Cancer (IARC)^15^.

Ni must be taken up by humans (adults – 45 µg/day) but is injurious when it is taken in excess. Humans are exposed to Ni for various reasons, such as ingestion of contaminated water, cigarette smoking, and contact with the skin from Ni-contaminated water or soil. Nickel sulfides and oxides have a greater risk of causing cancer^24^. It is known that Ni ions have a lower binding affinity for DNA and combine with histones present in the cell nucleus. Ni-nucleic acid histone complexes that are formed cause the initiation of DNA damage. They initiate carcinogenesis by attaching to DNA protein (histone), causing DNA to mutilate, leading to cross-linking of DNA interstrand and DNA proteins and breakage of the DNA strand^25^.

Chromium, especially Cr (III), is a nutrient that acts along with insulin to metabolize glucose, fat, and proteins (900 µg/day). The carcinogenic compounds are zinc chromate, calcium chromate, lead chromate, and strontium chromate, which are all hexavalent chromium compounds. The most carcinogenic form of chromium is CrO_4_ ^− 2^. They initiate carcinogenesis by producing reactive oxygen species (ROS) and suppressing the p53 gene^15^. The p53 gene is a tumour suppressor gene that plays a major role in cell division, and DNA repair, and inhibits angiogenesis. They function through transcription-mediated apoptosis and G1 cell cycle arrest^26^. Cr was present in our analysis, and Cr compounds cannot be assessed using LIBS, which was a limitation of our study.

Dhaware et al.^27^ determined the presence of toxic metals such as As, Cd, Cu, and Pb in various types of Indian smokeless tobacco (gutkha, creamy stuff, khaini, mishri, tooth powder, zarda) products using differential pulse anodic stripping voltammetry (DPASV). There were also negligible concentrations of other elements, such as Ni, Cr, and Hg. Mohammed et al.^28^ quantified the presence of various elements in Shisha and Doksha tobacco products using energy-dispersive X-ray fluorescence (EDXRF). They observed higher concentrations of calcium and other elements, such as Al, Ni, Cr, Fe, Mg, K, Zn, and strontium. Our study showed that all the commercially packaged forms of tobacco have the presence of carcinogenic elements such as Ni and other elements such as Cr, Fe, and Al were also observed. Patients who chewed various smokeless tobacco and smoked tobacco products with confirmed OSMF were assessed by Bagewasi et al.^29^ and Kode et al.^30^ to determine the role of trace elements in the etiopathogenesis of OSMF. They also reported that there was an increase in salivary copper levels in patients with OSMF who chewed various tobacco products. There was also an alteration in the Cu/Zn ratio and decreased Fe levels in patients with OSMF.

Surprisingly, there was no difference in the qualitative elemental composition of commercially packaged tobacco and ‘paan masala’ packets (which claimed no tobacco) in our study. It is true that our current attempt does not provide any leads to the quantitative assessment of these heavy metals. But with the LIBS technique, which has a detection limit of ppm levels (on direct sample analysis) in general, it was able to detect these elements, hinting at the abundance of these heavy metals in their content. We agree that these are indirect assumptions on the quantitative analysis part that should be verified using calibration-based/ calibration-free approaches in LIBS in future studies of the work.

## Conclusion

The consumption of smokeless forms of tobacco has increased over the past decade in India and Southeast Asia. Packaged forms of tobacco and betel quid are popularly consumed in India. Our study revealed the presence of various elements, such as calcium, aluminium, iron, chromium, and the known carcinogenic element nickel. Interestingly, the commercially packaged form (Paan masala), claimed by the manufacturer to be tobacco-free, has a similar composition to that of tobacco-containing packets. Recently, paan masala has gained more popularity over smokeless forms of tobacco owing to numerous commercial advertisements endorsing its use as a mouth freshener. This seems to have led people to believe paan masala to be harmless. Our study will hopefully generate debate amongst policymakers to relook at the potentially detrimental effects of paan masala and if required make laws regulating the sale and consumption of these products as well. Further, we recommend an assessment of the elemental composition of blood, saliva, and tissue specimens in patients with deleterious chewing habits. We hope that this study will provide insight into the role of elements involved in the etiopathogenesis of OPMDs and oral cancer.

## Data Availability

The datasets generated and analysed during the current study are available from the corresponding author upon reasonable request.
